# Lipoprotein(a): An important piece of the ASCVD risk factor puzzle across diverse populations

**DOI:** 10.1016/j.ahjo.2023.100350

**Published:** 2023-11-24

**Authors:** Nicole Ciffone, Catherine J. McNeal, Mary P. McGowan, Keith C. Ferdinand

**Affiliations:** a Arizona Center for Advanced Lipidology, 3925 E Fort Lowell Rd, Tucson, AZ 85712, USA; b Baylor Scott and White Health, 2401 S 31st St, Temple, TX 76508, USA; c The Family Heart Foundation, 680 E. Colorado Blvd, Suite 180, Pasadena, CA 91101, USA; d Dartmouth Hitchcock Medical Center, Geisel School of Medicine at Dartmouth, 1 Rope Ferry Rd, Hanover, NH 03755, USA; e John W. Deming Department of Medicine, Tulane University School of Medicine, 1430 Tulane Avenue, New Orleans, LA 70112, USA

**Keywords:** ASCVD, Atherogenic, Cardiovascular risk, Genetic, Lipoprotein(a), Testing

## Abstract

Elevated lipoprotein(a) (Lp[a]) is an independent, genetic risk factor for atherosclerotic cardiovascular disease (ASCVD) that impacts ~1.4 billion people globally. Generally, Lp(a) levels remain stable over time; thus, most individuals need only undergo Lp(a) testing through a non-fasting blood draw once in their lifetime, unless elevated Lp(a) is identified. Despite the convenience of the test for clinicians and patients, routine Lp(a) testing has not been widely adopted. This review provides a guide to the benefits of Lp(a) testing and solutions for overcoming common barriers in practice, including access to testing and lack of awareness. Lp(a) testing provides the opportunity to reclassify ASCVD risk and drive intensive cardiovascular risk factor management in individuals with elevated Lp(a), and to identify patients potentially less likely to respond to statins. Moreover, cascade screening can help to identify elevated Lp(a) in relatives of individuals with a personal or family history of premature ASCVD. Overall, given the profound impact of elevated Lp(a) on cardiovascular risk, Lp(a) testing should be an essential component of risk assessment by primary and specialty care providers.

## Highlights

• Elevated Lp(a) is an independent, genetic, and causal risk factor for ASCVD.
• Early detection of elevated Lp(a) may allow intensive CV risk factor management.
• Early identification of elevated Lp(a) may help reclassify ASCVD risk.
• All adults should undergo Lp(a) testing using a routine blood draw.
• Cascade screening can identify elevated Lp(a) in relatives.

## Graphical abstract

**Figure:**
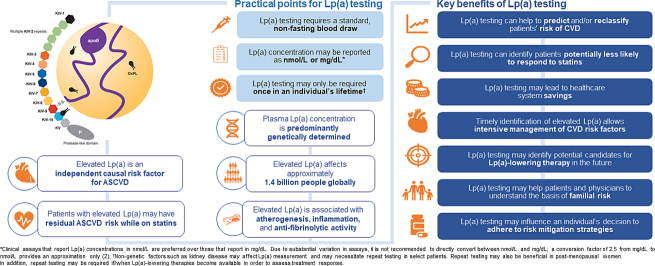

## 1 Introduction

Despite advances in diagnosing and treating cardiovascular disease (CVD), approximately 30 % of annual deaths worldwide remain attributable to CVD [1]. In addition to traditional risk factors, elevated lipoprotein(a) (Lp[a]) is a strong independent risk factor for the development of atherosclerotic cardiovascular disease (ASCVD) and affects approximately 1.4 billion people globally [[2], [3], [4], [5], [6]]. Elevated Lp(a) is also associated with cardiovascular (CV) and all-cause mortality [2,7,8].

Multiple medical societies recommend Lp(a) testing in individuals with a family history of premature ASCVD and/or elevated Lp(a) (Table 1) [2,[8], [9], [10], [11], [12]]. Moreover, the 2022 European Atherosclerosis Society (EAS) and the 2021 Canadian Cardiovascular Society guidelines recommend testing Lp(a) at least once in all adults, regardless of family history [2,11]. Given the potentially life-threatening effects of elevated Lp(a), there is a clear rationale to include an Lp(a) test for all patients undergoing lipid testing [13].

**Table 1:** Medical society and association recommendations for Lp(a) testing.

Society/association	Recommendations
AACE/ACE [9]	Test Lp(a) in the following populations: • All patients with clinical ASCVD or a family history of premature ASCVD and/or elevated Lp(a) • Patients with South Asian or African ancestry • Individuals with a 10-year ASCVD risk ≥10 % (primary prevention) • Patients with a personal or family history of AVS or those with refractory elevations of LDL-C despite aggressive LDL-C-lowering therapy

NLA [8]	Lp(a) testing is reasonable to refine risk assessment for ASCVD events in adults with: • First-degree relatives with premature ASCVD (<55 years of age in men; <65 years of age in women) • A personal history of premature ASCVD • Primary severe hypercholesterolemia (LDL-C ≥190 mg/dL) or suspected FH Lp(a) testing may be reasonable in adults: • To aid in the clinician-patient discussion on whether to prescribe a statin in those 40 to 75 years of age with borderline (5 to 7.4 %) 10-year ASCVD risk • To identify a possible cause for a less-than-anticipated LDL-C lowering to evidence-based LDL-C-lowering therapy • To use in cascade screening of family members with severe hypercholesterolemia • To identify those at risk for progressive AVS Lp(a) testing may be reasonable in youths (<20 years of age) with: • Clinically suspected or genetically confirmed FH • A family history of first-degree relatives with premature ASCVD (<55 years of age in men; <65 years of age in women) • An unknown cause of ischemic stroke • A parent or sibling found to have an elevated Lp(a)

AHA/ACC/Multi-society [10]	• Test Lp(a) in patients with a family history of premature ASCVD

EAS [2]	• Test Lp(a) concentration at least once in adults • Cascade testing may have value in FH, or patients with a history of very high or high Lp(a), or premature ASCVD

ESC/EAS [12]	• A one-off measurement of Lp(a) may help to identify people with very high inherited Lp(a) who may have a substantial lifetime risk of ASCVD

HEART UK [17]	Serum lipoprotein(a) levels should be measured in those with the following: • A personal or family history of premature ASCVD (<60 years of age) • First-degree relatives with raised serum Lp(a) (>200 nmol/L) • FH or other genetic dyslipidemias • Calcific AVS • Borderline increased (<15 %) 10-year risk of a cardiovascular event

CCS [11]	• Test Lp(a) concentration once in a person's lifetime as a part of initial lipid screening

Abbreviations: AACE, American Association of Clinical Endocrinologists; ACC, American College of Cardiology; ACE, American College of Endocrinology; AHA, American Heart Association; ASCVD, atherosclerotic cardiovascular disease; AVS, aortic valve stenosis; CCS, Canadian Cardiovascular Society; EAS, European Atherosclerosis Society; ESC, European Society of Cardiology; FH, familial hypercholesterolemia; LDL-C, low-density lipoprotein cholesterol; Lp(a), lipoprotein(a); NLA, National Lipid Association.

In this review, we outline clinical scenarios relating to elevated Lp(a) and provide a concise, practical guide on the key benefits of Lp(a) testing with proposed solutions for overcoming common barriers to testing in practice.

## 2 Lp(a) testing in practice: the status quo

**Fig. 1:**
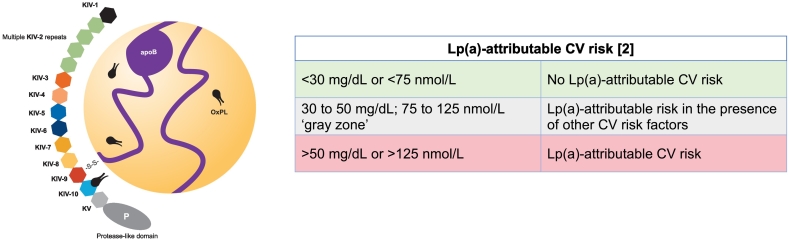
Structure of Lp(a), and Lp(a)-attributable ASCVD risk categories [2]. apoB – apolipoprotein B100, CV – cardiovascular, KIV – kringle IV, KV – kringle V, Lp(a) – lipoprotein(a), OxPL – oxidized phospholipid, P – protease; s-s – disulfide.

While low-density lipoprotein cholesterol (LDL-C) concentration is influenced by both genetics and lifestyle [14], the plasma concentration of Lp(a) is predominantly genetically determined (Table 2) [[2], [3], [4]]. The unique physiology of Lp(a) dictates its role in the pathogenesis of CVD. Lp(a) is an apolipoprotein B100 (apoB)-containing protein with a single apolipoprotein(a) (apo(a)) molecule and oxidized phospholipids (OxPLs) covalently bound to apo(a) (Fig. 1) [[15], [16], [17]].

**Table 2:** Lp(a): an ASCVD risk factor.

Can you recall patients who presented with premature CVD, were young, seemingly fit, and healthy, had never smoked, and were physically active?

This seemingly healthy patient may have elevated Lp(a), an independent genetic risk factor for ASCVD, which increases the risk of developing atherosclerosis when combined with other risk factors. Individuals with elevated Lp(a) also have a higher risk of aortic valve calcification and aortic valve stenosis compared with individuals with normal Lp(a) levels [8,24].

Elevated Lp(a) is associated with an increased risk of coronary heart disease, MI, stroke, and peripheral arterial disease [13,25]. Patients with elevated Lp(a) are potentially less likely to respond to statins, i.e., reduced LDL-C lowering [2,26,27], and will have residual ASCVD risk while on treatment with statins, regardless of LDL-C status [7,15,27,28].

Given that elevated Lp(a) affects approximately 20 % of the global population [29], it is likely that many of the patients we treat have elevated Lp(a) and are currently unaware. We know that elevated Lp(a) is inversely correlated with life expectancy [30] and, in most cases, is not identified until after a CV event has occurred. It is imperative that, as clinicians, we change the status quo, advocate for routine Lp(a) testing, and improve outcomes for patients with elevated Lp(a).

Abbreviations: ASCVD, atherosclerotic cardiovascular disease; CV, cardiovascular; CVD, cardiovascular disease; LDL-C, low-density lipoprotein cholesterol; Lp(a), lipoprotein(a); MI, myocardial infarction.

Lp(a) carries cholesterol, which is associated with atherogenic effects [18]. The apo(a) component of Lp(a) appears to have prothrombotic or antifibrinolytic activity [17], and the OxPLs bound to Lp(a) are key mediators of the pro-inflammatory and plaque-destabilizing actions of the particle [19]. It should be noted that Lp(a) and LDL are distinct members of the family of apoB-containing lipoproteins; therefore, Lp(a) can be elevated irrespective of LDL-C levels, [8] and Lp(a) levels cannot be inferred from other lipid levels such as LDL-C.

High Lp(a) levels are associated with accelerated progression of low-attenuation atherosclerotic plaques in patients with coronary artery disease, which may be associated with residual risk of myocardial infarction (MI) despite treatment with lipid-lowering therapy [20]. In addition, recent data from Rosenson et al., show that Lp(a) activates monocytes to drive inflammation and thrombosis, suggesting that elevated Lp(a) may directly impact increasing immunothrombotic risk in patients with ASCVD [21]. There are profound differences in Lp(a) levels among the global population, partly due to the varied distribution of plasma Lp(a) concentration among different racial/ethnic groups [8,22]. Studies indicate that median Lp(a) levels increase sequentially among Chinese, White, South Asian, and Black individuals [2]. Evidence also suggests that apo(a) isoform size can impact Lp(a) levels such that individuals with large apo(a) isoforms have significantly lower Lp(a) levels than those with small apo(a) isoforms [23]. Therefore, unlike other plasma lipoproteins such as apoB, Lp(a) does not have a single defined mass [13].

The occurrence of a premature ASCVD event, or recurrent events, despite intensive risk factor management, serves as a stimulus for ordering an Lp(a) test. A review of electronic health records from a large US database showed that, compared with individuals who underwent LDL-C testing alone, those who underwent Lp(a) testing were more likely to have experienced premature MI, ischemic stroke, and multiple prior CV events [31]. In clinical practice, adoption of Lp(a) testing is low, with rates of only 0.3 % among >112 million individuals screened or treated for ASCVD between 2012 and 2019 in the US Family Heart Database [32], and 13.9 % in 48,135 patients with diagnosed ASCVD in the global Lp(a)HERITAGE study [33]. This may be driven by the absence of approved therapies and a perceived inability to manage Lp(a)-associated ASCVD risk effectively.

Given the multiple pathogenic effects of elevated Lp(a), patients must be identified as early as possible to ensure more intensive CVD risk factor management, lifestyle modification, and/or pharmacologic intervention [2,4,5,34]. At present, high-intensity statins, and non-statin lipid-lowering therapies are indicated in lipid management guidelines to reduce CVD risk in the absence of approved Lp(a)-lowering therapies [2,8,9]. In clinical trials, anti-proprotein convertase subtilisin/kexin type 9 (PCSK9) monoclonal antibodies and a small interfering RNA that targets the production of PCSK9 reduced plasma Lp(a) by up to 27 % [[35], [36], [37], [38]]. However, since these trials were designed to evaluate the effects of LDL-C lowering, and median Lp(a) levels were relatively low, further studies on the potential Lp(a)-lowering effects of currently available lipid-lowering therapies are required.

## 3 Lp(a) testing: the ideal scenario

A 2019 Scientific Statement from the National Lipid Association (NLA), which was republished in 2022, highlights the importance of identifying elevated Lp(a) in young people to encourage early adoption of a heart-healthy lifestyle to decrease lifetime CVD risk, even in the absence of approved Lp(a)-lowering therapies [8]. In children, measurement of Lp(a) levels could be incorporated into the schedule for lipid testing outlined by the American Academy of Pediatrics (AAP). The AAP recommends that children with a family history of premature ASCVD or familial hypercholesterolemia (FH) should undergo their first lipid panel at 2 years of age [39]. The 2011 National Heart, Lung, and Blood Institute (NHLBI) integrated guideline on Cardiovascular Health and Risk Reduction in Children and Adolescents recommends that a lipid panel should be performed in children of 1 to 4 years of age if they have a family history of CVD or a parent with dyslipidemia [40]. The NHLBI guidelines also recommend universal lipid testing between 9 and 11 years of age in all children, regardless of family history [39].

Given that the *LPA* gene is transmitted in an autosomal codominant pattern, and an allele is inherited from each parent, cascade screening can help to identify elevated Lp(a) among close relatives of patients known to have elevated Lp(a) (Table 3) [1]. One study showed that systematic testing for elevated Lp(a) in relatives of patients with FH identified one new case of elevated Lp(a) for every 2.4 relatives screened [41].

**Table 3:** Lp(a) testing: the ideal scenario.

Adult levels of Lp(a) are present in children, sometimes by 2 years of age [42]; therefore, Lp(a) testing in youth, and particularly in childhood, is a feasible strategy that may help to modify ASCVD risk compared with patients who are tested later in life [30].

Awareness of elevated Lp(a) can equip parents with the knowledge needed to foster beneficial lifestyle choices as children grow into adults; these include lifelong tobacco avoidance, regular physical activity, and maintaining a healthy body weight [30].

In primary care, clinicians play a key role in recognizing family history of elevated cholesterol and premature heart disease and should aim to support cascade screening in extended family members of patients with elevated Lp(a) to enable risk mitigation in those identified as having elevated Lp(a) [43].

Abbreviations: ASCVD, atherosclerotic cardiovascular disease; Lp(a), lipoprotein(a).

Another study showed that among 182 relatives of patients with elevated Lp(a) who were tested, including 31 children, 68.1 % were found to have elevated Lp(a) (≥50 mg/dL [125 nmol/L]); 32.9 % had Lp(a) levels of 50 to 99 mg/dL (125 to 250 nmol/L); and 35.2 % had Lp(a) levels ≥100 mg/dL (250 nmol/L) [44]. These studies demonstrate the value of cascade screening for identifying individuals with elevated Lp(a), a key component of a preventive strategy to reduce their risk of developing ASCVD.

## 4 Key benefits of Lp(a) testing

**Fig. 2:**
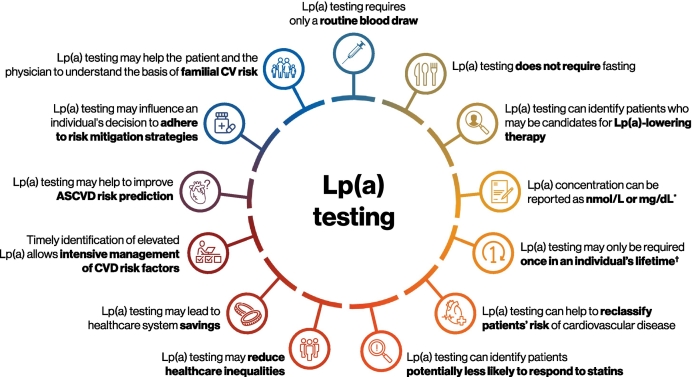
Summary of the key benefits of Lp(a) testing and practical points [1,2,5,17,26]. ASCVD – atherosclerotic cardiovascular disease, CV – cardiovascular, CVD – cardiovascular disease, Lp(a) – lipoprotein(a). *Clinical assays that report Lp(a) concentrations in nmol/L are preferred over those that report in mg/dL. Due to substantial variation in assays, it is not recommended to directly convert between nmol/L and mg/dL; a conversion factor of 2.5 from mg/dL to nmol/L provides an approximation only [2]; ^†^Non-genetic factors such as kidney disease may affect Lp(a) measurement and may necessitate repeat testing in select patients. Repeat testing may also be beneficial in post-menopausal women. In addition, repeat testing may be required if/when Lp(a)-lowering therapies become available in order to assess treatment response.

The key benefits of Lp(a) testing are summarized in Fig. 2.

### 4.1 Improved risk stratification

A key benefit of Lp(a) testing is the potential to improve CVD risk stratification, particularly in individuals with very high Lp(a) [2,26,45]. A study of patients who underwent lipid profiling in the Netherlands reported that 63 % of those with Lp(a) concentrations above the 99th percentile (>387.8 nmol/L) were reclassified into a higher CVD risk category based on the Second Manifestations of ARTerial disease (SMART) risk score [26]. Lp(a) testing also improves CVD risk prediction and enables reclassification of individuals at intermediate risk [45].

Data from the European Prospective Investigation into Cancer in Norfolk study of >14,000 participants showed that among patients with elevated Lp(a), those with ideal CV health had a significantly lower risk of CV events compared with those with poor CV health (relative risk 0.33, 95 % confidence interval [CI] 0.17–0.63; *P* < 0.001) [4]. The classification of ideal CV health in this study was based on seven risk factors, prioritized by the American Heart Association (AHA), that can be proactively managed to reduce CVD risk in patients with elevated Lp(a): body mass index, healthy diet, physical activity, smoking status, blood pressure, diabetes, and cholesterol levels [4].

Several tools are available for the prediction of CVD risk, including the Framingham risk score, American College of Cardiology/AHA ASCVD pooled cohort equations, and Systemic Coronary Risk Evaluation (SCORE) [46]. Lp(a) is not currently included in these tools but is considered a risk-modifying factor [2,26]. Encouragingly, a 2022 statement from the AHA provides guidance on how to incorporate Lp(a) concentration into a patient's 10-year CVD risk estimate, based on the following formula: predicted 10-year risk×(1.11[patients Lp(a) level in nmol/L/50]) [3]. Of note, the true concentration of LDL-C is often lower than recognized in patients with elevated Lp(a), since a portion of plasma cholesterol can be carried by Lp(a) particles, i.e. in patients with an elevated Lp(a) protein level, the calculated LDL-C includes Lp(a)-cholesterol [5]. Therefore, even at very low LDL-C concentrations, elevated Lp(a) is an important risk factor for CVD. [2]

Adopting widespread Lp(a) testing can help generate data on the real-world prevalence of elevated Lp(a), which may contribute to the refinement of CV risk assessment and the definition of clinically relevant risk thresholds [1,17]. This may be important for individuals from differing racial/ethnic backgrounds; e.g. median Lp(a) levels among African American adults are approximately 3-fold higher than in White individuals (75 nmol/L vs. 20 nmol/L) [8]. Consequently, an Lp(a) level > 100 nmol/L corresponds to the 80th percentile in White individuals, while the 80th percentile in people from an African American background is around 150 nmol/L. Importantly, however, recent studies suggest that the predictive value of Lp(a) may not vary sufficiently to support a need for race-specific thresholds in clinical decision-making [47,48]. Evidence is also incomplete regarding the use of differing risk thresholds according to age, sex/gender, or comorbidities [8]. Given the uncertainty surrounding the use of distinct risk thresholds, collaborations between medical societies and policymakers are needed to provide detailed recommendations for clinical practice [7].

### 4.2 Cost-effectiveness

Another key benefit associated with Lp(a) testing is the potential to facilitate cost-effective use of healthcare resources through early diagnosis and optimal management of individuals with elevated Lp(a) [44]. In addition, given that Lp(a) testing is likely to be required only once in a lifetime for adult individuals with normal levels, the cost of testing may be offset by reduced healthcare utilization in patients with elevated Lp(a) who can intensively manage their CVD risk factors [1,13]. As such, the cost-benefit ratio of Lp(a) testing versus the potential for lowering the future financial burden associated with premature ASCVD warrants further assessment [42].

## 5 Practical considerations for Lp(a) testing

Practical points associated with Lp(a) testing are provided in Fig. 2. It is important to note that an Lp(a) test requires only a routine, non-fasting blood draw [3,26,42,49].

Lp(a) concentrations are generally considered to vary by <10 % over time in any individual, and a single lifetime assessment of Lp(a) may be suitable for most people [1,50,51]. Nonetheless, a recent study reported that in some individuals, Lp(a) levels varied by approximately 20 % over 190 days [52]. Some non-genetic factors may affect Lp(a) measurement, including kidney disease, acute infection, and pregnancy, which may necessitate repeat testing in select patients [1,2]. A second Lp(a) test may also benefit post-menopausal women, since Lp(a) levels increase following menopause [2,53].

## 6 Proposed solutions to common barriers to Lp(a) testing

**Fig. 3:**
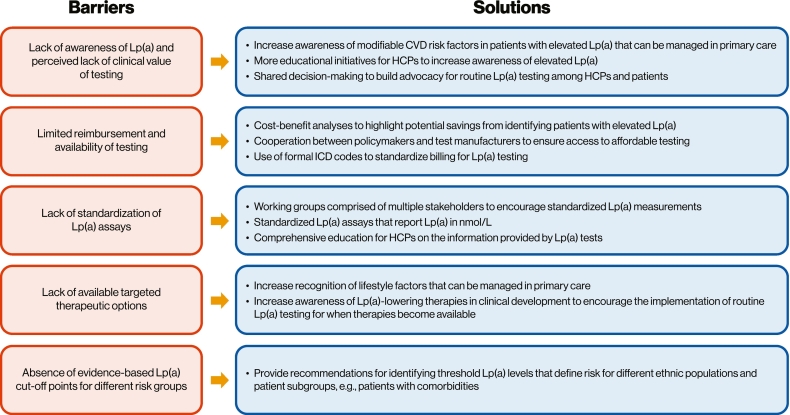
Proposed solutions for common barriers to Lp(a) testing [1,2]. CVD – cardiovascular disease, HCP – healthcare professional, ICD – International Classification of Diseases, Lp(a) – lipoprotein(a).

Key obstacles to Lp(a) testing and proposed solutions are summarized in Fig. 3. The foundation of these proposed solutions is collaboration between multiple stakeholders, including clinicians, patients, and policymakers, with the aim of implementing routine Lp(a) testing in clinical practice.

### 6.1 Increasing awareness of Lp(a) and the clinical value of testing

The 2022 EAS Consensus Statement on Lp(a) in ASCVD and aortic stenosis proposes a model of care that incorporates the importance of Lp(a) screening and ASCVD risk stratification alongside the need for educational initiatives that foster behavioral change and wider implementation of Lp(a) testing in practice [2]. As part of these initiatives, the EAS recommends several strategies for engaging with patients, including social media and educational webinars [2]. The NLA also recognizes the importance of patient engagement and advises that the decision to test for Lp(a) should be made after a comprehensive benefit-risk discussion that considers family history, comorbidities, and patients' concerns surrounding the future risk of CV events [8]. Knowledge of Lp(a) levels in individuals with a family history of premature CVD (<60 years of age) may help the patient and physician to understand the basis of the familial CV risk and influence an individual's decision to start and adhere to long-term medication [17].

As clinicians, we can engage in shared decision-making with our patients and utilize patient-focused educational materials to improve patients' knowledge of the risks associated with elevated Lp(a) and the importance of Lp(a) testing [54]. Patients can be directed to societies or advocacy groups, such as the Family Heart Foundation (https://familyheart.org/), which offers frequent patient- and clinician-facing webinars on Lp(a), for more information, particularly with respect to family screening [55].

### 6.2 Expanding access to Lp(a) testing

Wider implementation of Lp(a) testing in clinical practice may be achieved, in part, through analyses of the potential cost savings associated with earlier identification and management of patients with elevated Lp(a). If clear savings can be demonstrated through robust data, guideline recommendations for Lp(a) testing may be more readily translated into clinical practice [1]. The advent of insurance codes for reimbursement of Lp(a) testing (International Classification of diagnosis [ICD]-10: E78.41 and Z83.430; Current Procedural Terminology [CPT®] 83,695) are also vital for expanding access [5,56,57]. Importantly, these codes encourage Lp(a) testing not only in individuals with premature CV events but also in seemingly healthy individuals, particularly those with a family history of elevated Lp(a) and/or premature ASCVD [56].

Policymakers can play a key role in expanding access to Lp(a) testing by incentivizing diagnostics manufacturers to develop Lp(a) assays for currently used platforms [1]. This should make testing more accessible on a larger scale and ensure that more individuals with elevated Lp(a) are identified. Increased adoption of routine Lp(a) testing may also help tackle healthcare inequalities and ensure access for individuals from all socioeconomic backgrounds, including those with lower socioeconomic status who are unlikely to request a test, especially if they must pay for it themselves [1].

### 6.3 Standardizing Lp(a) assays

Lp(a) levels can be reported in mg/dL or nmol/L and should be measured using an assay that is isoform-insensitive and reported in molar units where possible, as this provides an accurate indication of Lp(a) concentration [51]. In contrast, methods that report Lp(a) levels in mg/dL are sensitive to the size of the apo(a) isoform and may report values that deviate from the real concentration [51]. Regardless of how Lp(a) test results are reported, high is high and steps should be taken to further assess the result to ensure intensive CVD risk factor management in all individuals with elevated Lp(a) [2].

Standardized reporting and training on the interpretation of Lp(a) test results will be vital for successful implementation [2]. A pioneering example is the Sulpizio Cardiovascular Center Lp(a) Clinic at the University of California in San Diego, which offers training opportunities for clinical fellows to review lipid profiles and interpret Lp(a) test results [5].

### 6.4 Overcoming additional barriers

One of the key barriers to implementing Lp(a) testing is the lack of approved targeted therapies that lower Lp(a). Several RNA-targeted Lp(a)-lowering therapies are now entering late-stage clinical development and have demonstrated significant reductions in Lp(a) levels in clinical studies [49]. Should these novel therapies prove to be well tolerated and effective at reducing ASCVD events, to realize their potential in the clinic fully we must advocate for the widespread adoption of Lp(a) testing to identify patients who may be eligible for pharmacologic treatment in the future [51,58,59]. This is especially pertinent given that policy interventions take time to implement and even longer to translate into a measurable change in clinical practice.

To ensure that patients are stratified according to their individual CV risk level, it will be important to confirm whether distinct risk thresholds are required for individuals from different ethnic backgrounds. This is also true for other groups, e.g. patients with comorbidities [7]; however, more studies are required.

## 7 Conclusions

Elevated Lp(a) is associated with an increased risk of ASCVD events, even in patients receiving lipid-lowering therapies who have achieved LDL-C goals [5,8]. Timely identification of elevated Lp(a) is vital for recognizing individuals who may benefit from interventions to reduce CV risk, including, as broadly agreed in guidelines and position statements, those with a family history of premature ASCVD [2,4]. In clinical practice, an Lp(a) test is usually only ordered after a premature CV event has occurred. Therefore, we believe it is imperative that close relatives of patients with elevated Lp(a), including children, be tested.

An Lp(a) test needs only a routine, non-fasting blood draw and usually only needs to be measured once in a lifetime in most adult individuals [26,42,49]. Unfortunately, despite advocacy from several medical societies for a single lifetime assessment of Lp(a) in all individuals and/or testing in relatives of patients with elevated Lp(a) to identify those with high CV risk [2,[8], [9], [10]], routine testing is not currently implemented in clinical practice [26]. Low adoption of Lp(a) testing may be driven by the absence of approved therapies; however, several RNA-targeted Lp(a)-lowering therapies are in clinical development and have shown significant reductions in Lp(a) levels [49]. In the meantime, strategies such as LDL-C-lowering and lifestyle modifications are recommended in individuals with elevated Lp(a) to reduce their overall CV risk [2]. We can support individuals with elevated Lp(a) to implement lifestyle modifications such as regular physical activity, smoking cessation, and heart-healthy and/or plant-based diets, which have all been proposed to mitigate CV risk in this population [34,60,61].

Overall, given the profound impact of elevated Lp(a) on CV risk, routine Lp(a) testing should be an essential component of lipid measurement in primary care and specialty practices, especially cardiology. The time is now to advocate for Lp(a) testing, maximize awareness of elevated Lp(a) as an independent CV risk factor among patients and fellow clinicians, and ultimately build a brighter future for patients with elevated Lp(a).

## Declaration of competing interest

The authors declare the following financial interests/personal relationships which may be considered as potential competing interests: Ciffone – Previous consulting: Novartis. McNeal – Previous consulting: Novartis. McGowan – Previous participation in a Novartis advisory board. Ferdinand – Consulting: Amgen, Janssen, Medtronic, and Novartis.
